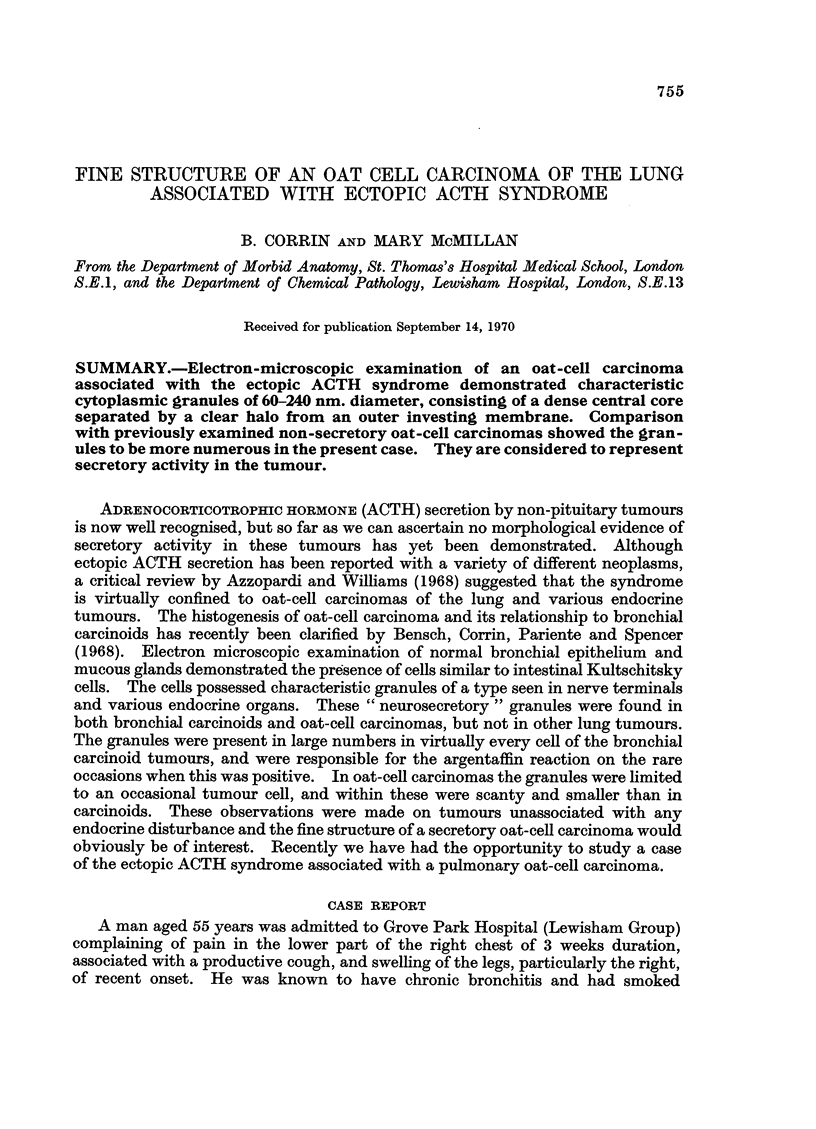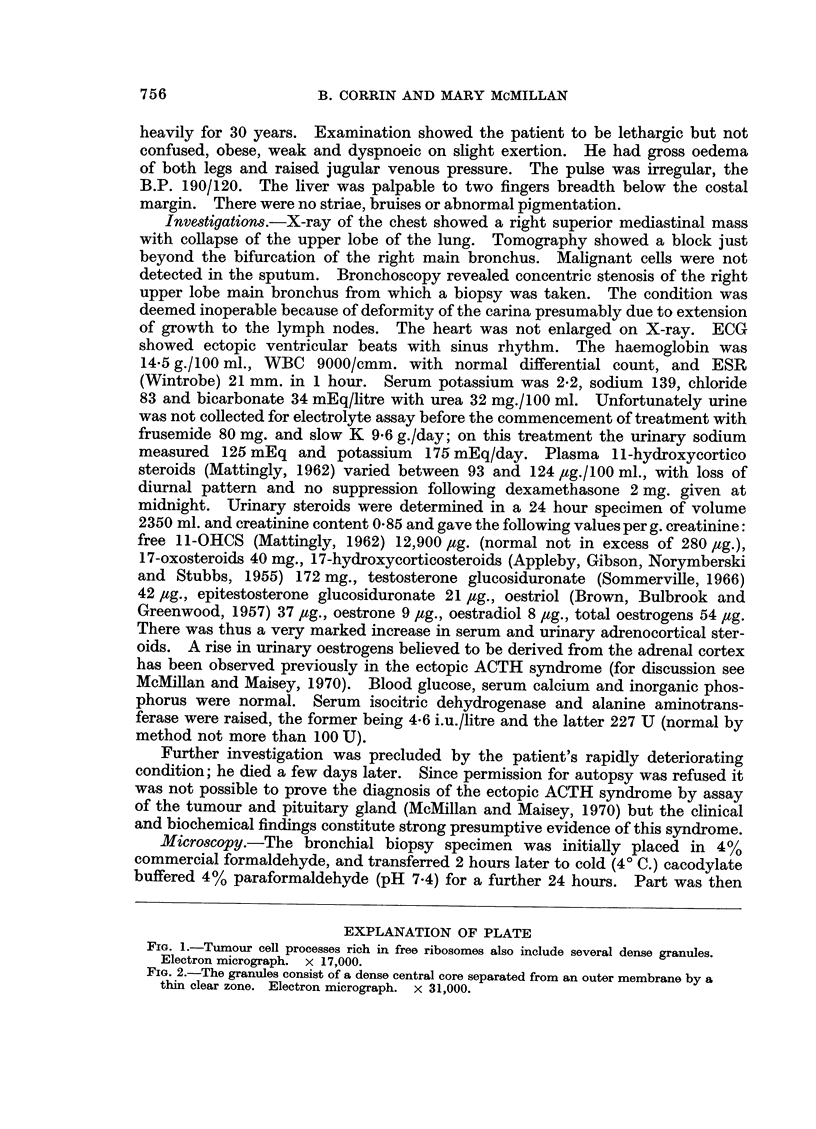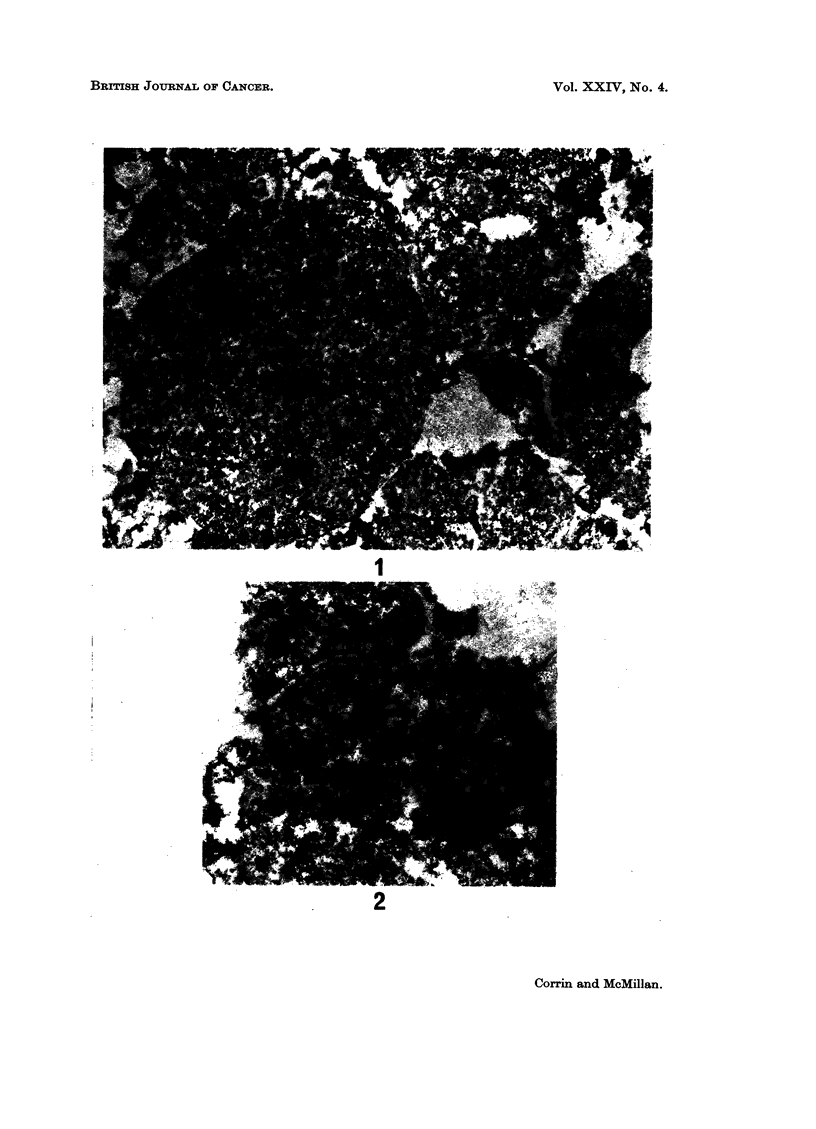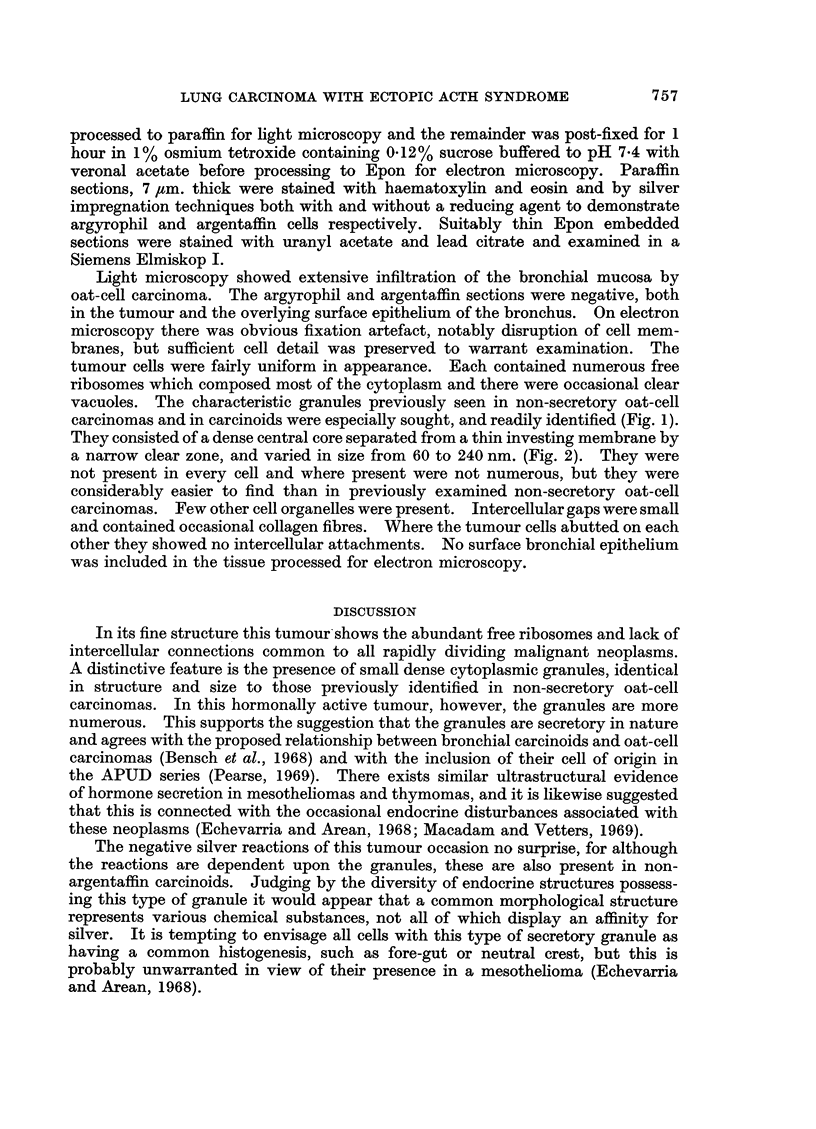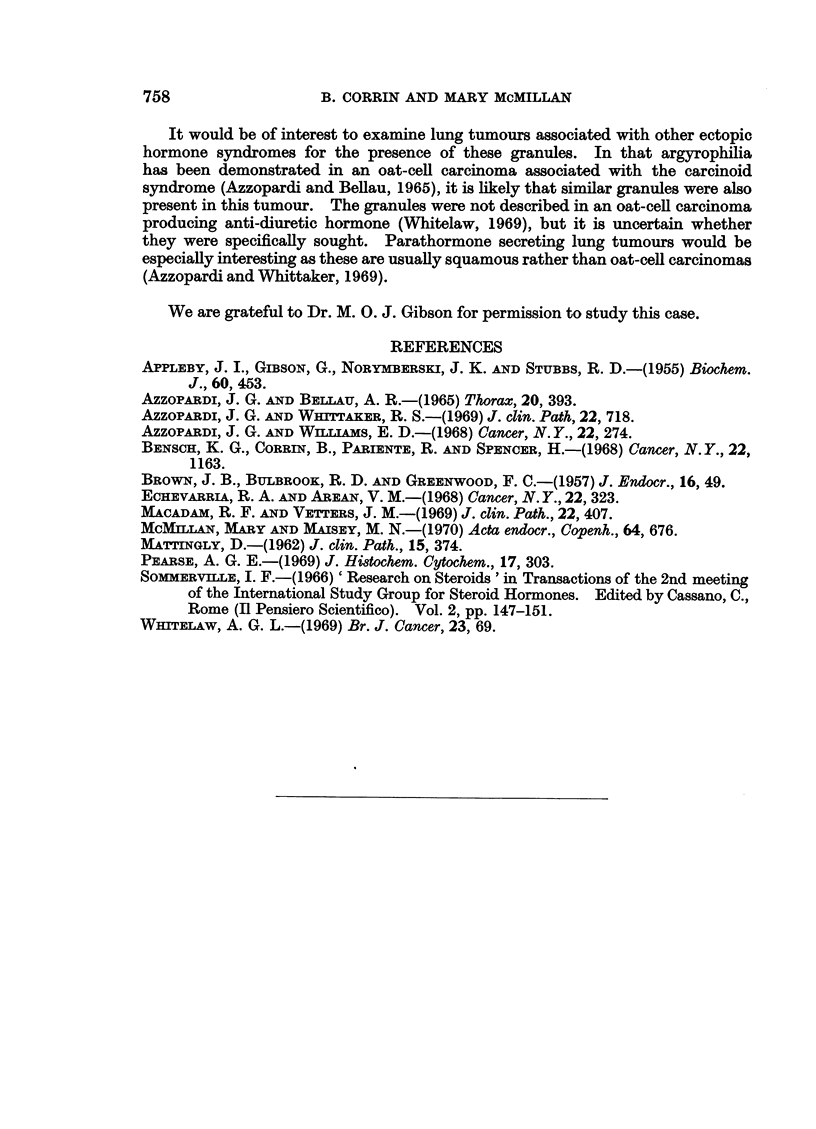# Fine Structure of an Oat Cell Carcinoma of the Lung Associated with Ectopic ACTH Syndrome

**DOI:** 10.1038/bjc.1970.90

**Published:** 1970-12

**Authors:** B. Corrin, Mary McMillan

## Abstract

**Images:**


### FINE STRUCTURE OF AN OAT CELL CARCINOMA OF THE LUNG ASSOCIATED WITH ECTOPIC ACTH SYNDROME

B. CORRIN AND MARY McMILLAN

*From the Department of Morbid Anatomy, St. Thomas's Hospital Medical School, London S.E.1, and the Department of Chemical Pathology, Lewisham Hospital, London, S.E.13*

Received for publication September 14, 1970

**SUMMARY.—Electron-microscopic examination of an oat-cell carcinoma associated with the ectopic ACTH syndrome demonstrated characteristic cytoplasmic granules of 60–240 nm. diameter, consisting of a dense central core separated by a clear halo from an outer investing membrane. Comparison with previously examined non-secretory oat-cell carcinomas showed the granules to be more numerous in the present case. They are considered to represent secretory activity in the tumour.**

ADRENOCORTICOTROPHIC HORMONE (ACTH) secretion by non-pituitary tumours is now well recognised, but so far as we can ascertain no morphological evidence of secretory activity in these tumours has yet been demonstrated. Although ectopic ACTH secretion has been reported with a variety of different neoplasms, a critical review by Azzopardi and Williams (1968) suggested that the syndrome is virtually confined to oat-cell carcinomas of the lung and various endocrine tumours. The histogenesis of oat-cell carcinoma and its relationship to bronchial carcinoids has recently been clarified by Bensch, Corrin, Pariente and Spencer (1968). Electron microscopic examination of normal bronchial epithelium and mucous glands demonstrated the presence of cells similar to intestinal Kultschitsky cells. The cells possessed characteristic granules of a type seen in nerve terminals and various endocrine organs. These " neurosecretory " granules were found in both bronchial carcinoids and oat-cell carcinomas, but not in other lung tumours. The granules were present in large numbers in virtually every cell of the bronchial carcinoid tumours, and were responsible for the argentaffin reaction on the rare occasions when this was positive. In oat-cell carcinomas the granules were limited to an occasional tumour cell, and within these were scanty and smaller than in carcinoids. These observations were made on tumours unassociated with any endocrine disturbance and the fine structure of a secretory oat-cell carcinoma would obviously be of interest. Recently we have had the opportunity to study a case of the ectopic ACTH syndrome associated with a pulmonary oat-cell carcinoma.

### CASE REPORT

A man aged 55 years was admitted to Grove Park Hospital (Lewisham Group) complaining of pain in the lower part of the right chest of 3 weeks duration, associated with a productive cough, and swelling of the legs, particularly the right, of recent onset. He was known to have chronic bronchitis and had smoked heavily for 30 years. Examination showed the patient to be lethargic but not confused, obese, weak and dyspnoeic on slight exertion. He had gross oedema of both legs and raised jugular venous pressure. The pulse was irregular, the B.P. 190/120. The liver was palpable to two fingers breadth below the costal margin. There were no striae, bruises or abnormal pigmentation.

*Investigations.*—X-ray of the chest showed a right superior mediastinal mass with collapse of the upper lobe of the lung. Tomography showed a block just beyond the bifurcation of the right main bronchus. Malignant cells were not detected in the sputum. Bronchoscopy revealed concentric stenosis of the right upper lobe main bronchus from which a biopsy was taken. The condition was deemed inoperable because of deformity of the carina presumably due to extension of growth to the lymph nodes. The heart was not enlarged on X-ray. ECG showed ectopic ventricular beats with sinus rhythm. The haemoglobin was 14·5 g./100 ml., WBC 9000/cmm. with normal differential count, and ESR (Wintrobe) 21 mm. in 1 hour. Serum potassium was 2·2, sodium 139, chloride 83 and bicarbonate 34 mEq/litre with urea 32 mg./100 ml. Unfortunately urine was not collected for electrolyte assay before the commencement of treatment with frusemide 80 mg. and slow K 9·6 g./day; on this treatment the urinary sodium measured 125 mEq and potassium 175 mEq/day. Plasma 11-hydroxycortico steroids (Mattingly, 1962) varied between 93 and 124 $\mu$g./100 ml., with loss of diurnal pattern and no suppression following dexamethasone 2 mg. given at midnight. Urinary steroids were determined in a 24 hour specimen of volume 2350 ml. and creatinine content 0·85 and gave the following values per g. creatinine: free 11-OHCS (Mattingly, 1962) 12,900 $\mu$g. (normal not in excess of 280 $\mu$g.), 17-oxosteroids 40 mg., 17-hydroxycorticosteroids (Appleby, Gibson, Norymberski and Stubbs, 1955) 172 mg., testosterone glucosiduronate (Sommerville, 1966) 42 $\mu$g., epitestosterone glucosiduronate 21 $\mu$g., oestriol (Brown, Bulbrook and Greenwood, 1957) 37 $\mu$g., oestrone 9 $\mu$g., oestradiol 8 $\mu$g., total oestrogens 54 $\mu$g. There was thus a very marked increase in serum and urinary adrenocortical steroids. A rise in urinary oestrogens believed to be derived from the adrenal cortex has been observed previously in the ectopic ACTH syndrome (for discussion see McMillan and Maisey, 1970). Blood glucose, serum calcium and inorganic phosphorus were normal. Serum isocitric dehydrogenase and alanine aminotransferase were raised, the former being 4·6 i.u./litre and the latter 227 U (normal by method not more than 100 U).

Further investigation was precluded by the patient's rapidly deteriorating condition; he died a few days later. Since permission for autopsy was refused it was not possible to prove the diagnosis of the ectopic ACTH syndrome by assay of the tumour and pituitary gland (McMillan and Maisey, 1970) but the clinical and biochemical findings constitute strong presumptive evidence of this syndrome.

*Microscopy.*—The bronchial biopsy specimen was initially placed in 4% commercial formaldehyde, and transferred 2 hours later to cold (4° C.) cacodylate buffered 4% paraformaldehyde (pH 7·4) for a further 24 hours. Part was then

---

EXPLANATION OF PLATE

FIG. 1.—Tumour cell processes rich in free ribosomes also include several dense granules. Electron micrograph. × 17,000.

FIG. 2.—The granules consist of a dense central core separated from an outer membrane by a thin clear zone. Electron micrograph. × 31,000.

1

2

Corrin and McMillan.

processed to paraffin for light microscopy and the remainder was post-fixed for 1 hour in 1% osmium tetroxide containing 0·12% sucrose buffered to pH 7·4 with veronal acetate before processing to Epon for electron microscopy. Paraffin sections, 7 $\mu$m. thick were stained with haematoxylin and eosin and by silver impregnation techniques both with and without a reducing agent to demonstrate argyrophil and argentaffin cells respectively. Suitably thin Epon embedded sections were stained with uranyl acetate and lead citrate and examined in a Siemens Elmiskop I.

Light microscopy showed extensive infiltration of the bronchial mucosa by oat-cell carcinoma. The argyrophil and argentaffin sections were negative, both in the tumour and the overlying surface epithelium of the bronchus. On electron microscopy there was obvious fixation artefact, notably disruption of cell membranes, but sufficient cell detail was preserved to warrant examination. The tumour cells were fairly uniform in appearance. Each contained numerous free ribosomes which composed most of the cytoplasm and there were occasional clear vacuoles. The characteristic granules previously seen in non-secretory oat-cell carcinomas and in carcinoids were especially sought, and readily identified (Fig. 1). They consisted of a dense central core separated from a thin investing membrane by a narrow clear zone, and varied in size from 60 to 240 nm. (Fig. 2). They were not present in every cell and where present were not numerous, but they were considerably easier to find than in previously examined non-secretory oat-cell carcinomas. Few other cell organelles were present. Intercellular gaps were small and contained occasional collagen fibres. Where the tumour cells abutted on each other they showed no intercellular attachments. No surface bronchial epithelium was included in the tissue processed for electron microscopy.

### DISCUSSION

In its fine structure this tumour shows the abundant free ribosomes and lack of intercellular connections common to all rapidly dividing malignant neoplasms. A distinctive feature is the presence of small dense cytoplasmic granules, identical in structure and size to those previously identified in non-secretory oat-cell carcinomas. In this hormonally active tumour, however, the granules are more numerous. This supports the suggestion that the granules are secretory in nature and agrees with the proposed relationship between bronchial carcinoids and oat-cell carcinomas (Bensch *et al.*, 1968) and with the inclusion of their cell of origin in the APUD series (Pearse, 1969). There exists similar ultrastructural evidence of hormone secretion in mesotheliomas and thymomas, and it is likewise suggested that this is connected with the occasional endocrine disturbances associated with these neoplasms (Echevarria and Arean, 1968; Macadam and Vetters, 1969).

The negative silver reactions of this tumour occasion no surprise, for although the reactions are dependent upon the granules, these are also present in non-argentaffin carcinoids. Judging by the diversity of endocrine structures possessing this type of granule it would appear that a common morphological structure represents various chemical substances, not all of which display an affinity for silver. It is tempting to envisage all cells with this type of secretory granule as having a common histogenesis, such as fore-gut or neutral crest, but this is probably unwarranted in view of their presence in a mesothelioma (Echevarria and Arean, 1968).

It would be of interest to examine lung tumours associated with other ectopic hormone syndromes for the presence of these granules. In that argyrophilia has been demonstrated in an oat-cell carcinoma associated with the carcinoid syndrome (Azzopardi and Bellau, 1965), it is likely that similar granules were also present in this tumour. The granules were not described in an oat-cell carcinoma producing anti-diuretic hormone (Whitelaw, 1969), but it is uncertain whether they were specifically sought. Parathormone secreting lung tumours would be especially interesting as these are usually squamous rather than oat-cell carcinomas (Azzopardi and Whittaker, 1969).

We are grateful to Dr. M. O. J. Gibson for permission to study this case.

### REFERENCES

Appleby, J. I., Gibson, G., Norymberski, J. K. and Stubbs, R. D.—(1955) *Biochem. J.*, **60**, 453.

Azzopardi, J. G. and Bellau, A. R.—(1965) *Thorax*, **20**, 393.

Azzopardi, J. G. and Whittaker, R. S.—(1969) *J. clin. Path*, **22**, 718.

Azzopardi, J. G. and Williams, E. D.—(1968) *Cancer, N.Y.*, **22**, 274.

Bensch, K. G., Corrin, B., Pariente, R. and Spencer, H.—(1968) *Cancer, N.Y.*, **22**, 1163.

Brown, J. B., Bulbrook, R. D. and Greenwood, F. C.—(1957) *J. Endocr.*, **16**, 49.

Echevarria, R. A. and Arean, V. M.—(1968) *Cancer, N.Y.*, **22**, 323.

Macadam, R. F. and Vetters, J. M.—(1969) *J. clin. Path.*, **22**, 407.

McMillan, Mary and Maisey, M. N.—(1970) *Acta endocr., Copenh.*, **64**, 676.

Mattingly, D.—(1962) *J. clin. Path.*, **15**, 374.

Pearse, A. G. E.—(1969) *J. Histochem. Cytochem.*, **17**, 303.

Sommerville, I. F.—(1966) ' Research on Steroids ' in Transactions of the 2nd meeting of the International Study Group for Steroid Hormones. Edited by Cassano, C., Rome (Il Pensiero Scientifico). Vol. 2, pp. 147–151.

Whitelaw, A. G. L.—(1969) *Br. J. Cancer*, **23**, 69.